# Seroprevalence study reveals pertussis underreporting in Brazil and calls for adolescent/young adult boosting: mouse model demonstrates immunity restoration

**DOI:** 10.3389/fimmu.2024.1472157

**Published:** 2024-12-04

**Authors:** Eliane P. Silva, Monalisa Trentini, Dunia Rodriguez, Alex I. Kanno, Filumena M. S. Gomes, Maria H. Valente, Carlos E. M. Trufen, Lais S. Yamamoto, Arthur D. Januzzi, Priscila S. Cunegundes, Ricardo Palacios, Renan P. Souza, Isaías Raw, Luciana C. C. Leite, Waldely O. Dias

**Affiliations:** ^1^ Laboratório de Desenvolvimento de Vacinas, Instituto Butantan, São Paulo, Brazil; ^2^ Department of Pediatrics, Faculdade de Medicina da Universidade de São Paulo, São Paulo, Brazil; ^3^ Clinical Trials Division, Instituto Butantan, São, Paulo, Brazil; ^4^ Instituto de Ciências Biológicas, Universidade Federal de Minas Gerais, Belo Horizonte, Brazil

**Keywords:** pertussis, seroprevalence, whole cell pertussis vaccine, acellular pertussis vaccine, pertussis low LPS vaccine

## Abstract

**Background:**

Pertussis continues to pose a significant threat despite the availability of effective vaccines. The challenge lies in the vulnerability of infants who have not yet completed their vaccination schedule and in adolescents and adults becoming potential disease carriers.

**Methods:**

We evaluated the seroprevalence of pertussis immunity in a cohort of 1,500 healthy Brazilian volunteers. Next, we explored the potential restoration of waning pertussis immunity by administering booster doses of wP, aP or Plow (an economically viable and low reactogenic vaccine in development at Butantan) using a mouse model.

**Findings:**

The mean anti-PT IgG levels in the Brazilian volunteers was 39.4 IU/mL. Notably, individuals ≤ 19 years exhibited higher IgG values compared to older age groups (≥ 20 y). Overall, 8.4% of the samples displayed indications of recent or current contact/infection, with IgG levels surpassing 120 IU/mL, particularly in the 15-19 years age group. IgM values were also increased in the 10-19 years age group. Potential recovery of pre-existing but waning immunity investigated in mice, showed that boosting with wP induced higher antibody titers than aP or Plow. Notably, aP and Plow boosts prompted superior effector and memory cell responses from both B and T cells. Upon challenge with *B. pertussis*, aP or Plow boost provided greater protection as compared to wP.

**Interpretations:**

Pertussis appears to circulate predominantly among adolescents and young adults. Insights from the mouse model indicate that immunity can be restored with booster doses. Boosting immunity in non-targeted groups could prevent the dissemination of pertussis to infants.

## 1 Introduction

Pertussis, or whooping cough, is a highly contagious respiratory infectious disease caused by *Bordetella pertussis*. Children under one year old are especially vulnerable, with 24 million cases and over 160,000 deaths/year worldwide (1). In Brazil, the whole cell pertussis vaccine (wP) is given as part of the pentavalent vaccine (diphteriae-tetanus-pertussis-*Haemophilus influenza* B and hepatitis B) at 2, 4 and 6 months of age. Booster doses with DTwP (diphteriae-tetanus-pertussis) are given at 1.5 and 4-5 years (2). Despite high vaccine coverage, pertussis is still circulating in the population, manifesting cyclically with peak incidence occurring every 2-5 years, as is typical for the disease. For example, between 2011 and 2015, the incidence (cases/100,000 inhabitants) peaked at 4.2 in 2014, up from previous 0.3 in 2010 (**Supplementary Table 1**). Another cycle occurred between 2016 and 2019, peaking again in 2018, with an incidence of 1.0 cases per 100,000 (**Supplementary Figure 1**).

Regarding vaccine coverage, there has been a steady decline since 2015. By 2019, only 71% of the targeted population received the pentavalent vaccine. Vaccination of pregnant women with the acellular pertussis vaccine (aP) began in 2014, using the dTpa vaccine aiming to protect newborns through passive antibody transfer. Vaccine coverage rapidly increased to 45% in 2015 but fluctuated between 34% (2016) and 63% (2019). While this additional vaccination may have contributed to the overall decrease in incidence, the resurgence of cases between 2016 and 2019 suggests that additional strategies may be necessary to fully control the disease (3).

There are two types of vaccines currently in use against pertussis: whole-cell (wP) and acellular vaccines (aP). While wP is considered more reactogenic, the high cost of aP limits its wide use in low- and middle-income countries (4). While both vaccines can induce specific antibodies, wP can better activate cellular T-helper 1 and 17 responses (5), which may explain wP’s longer-lasting protection. Loss of protection occurs four to 12 years after the last vaccine dose (6) and there is evidence that aP-induced protection wanes faster than wP (6). Furthermore, it has been demonstrated that aP does not prevent transmission of the bacteria, which can perpetuate continuous circulation of *Bordetella* in the vaccinated population (7, 8) Additionally, with vaccination strategies focusing on infants, adolescents and young adults may become reservoirs from which pertussis may spread to susceptible children (9). While infections in neonates are usually overtly symptomatic leading to medical attention, infected adolescents and adults often exhibit mild cold-like symptoms that are often ignored. Such differences significantly impact long-term pertussis control and contribute to its persistence despite decades of continuous immunization programs.

Integrating adolescent or young adult booster doses could reduce pertussis dissemination. Additionally, reactogenicity issues with wP, cost and faster waning immunity with aP highlight the need for improved vaccines. Plow vaccine has been developed at Instituto Butantan (10). It is a whole-cell vaccine with lower reactogenicity, obtained by extracting the outer membrane lipopolysaccharides (LPS). In a phase I comparative trial in new-borns, Plow demonstrated comparable immunogenicity and lower reactogenicity than wP (11). Plow represents a potential low-cost/low reactogenic pertussis vaccine alternative.

This study investigated pertussis immunity seroprevalence in a healthy population in Brazil. The results allowed us to raise a hypothesis, which was preliminarily investigated in mice: whether a waning pre-existing immunity could be restored by wP, aP or Plow boosters, ultimately to prevent its dissemination to higher-risk groups.

## 2 Materials and methods

### 2.1 Human blood collection

1,500 individuals were enrolled in the study. Blood samples were collected between May 2015 and October 2018 in São Paulo city at the Hospital Universitário of Universidade de São Paulo (HU/USP) in São Paulo. There was no formal sample size calculation, but we used the predicted incidence to have sufficient samples in each age group to achieve significance in their comparison (~100 – 200 in each group); our goal was to obtain 1.500 samples. In this time period there were many more samples from the 20-59 groups. The number of samples collected each year were as follows: 2015 (n=540), 2016 (n=495), 2017 (n=400) and 2018 (n=65). Exclusion criteria were medical record of tuberculosis, immunodeficiencies or neoplasia; pregnant, breast-feeding woman and people under antibiotic treatment, conditions that could interfere with the results.

Heparinized blood and serum were used in the assays. 10 mL of blood was collected in Vacutainer^®^ (Becton-Dickinson, BD) heparinized tubes (for stimulation and cytokine assessment) and 4 mL in another Vacutainer^®^ tube containing a clot activator and serum gel separator (for antibody detection). Serum samples were separated by centrifugation, individually identified, and stored at -20°C until use.

For determination of anti-PT IgG, 1,500 serum samples were stratified in age groups. As we had access to samples from donors 4 y and older, they were grouped in the 4-9 y age group. The number of serum samples collected from each group was allocated as follows: 4-9 (n=159), 10-14 (n=129), 15-19 (n=94), 20-39 (n=425), 40-59 (n=465) and ≥ 60 (n=228). Of these, 1,024 samples were analyzed for determination of anti-PT IgM divided as follows: 4-9 (n=125), 10-14 (n=90), 15-19 (n=65), 20-39 (n=295), 40-59 (298), ≥ 60 (n=151) (**Table 1**).

**Table 1:** Distribution by age group/gender of blood samples from volunteers collected between 2015-2019 – HU/USP – São Paulo.

Age groups (years)	Gender		N° of volunteers	Fully vaccinated (%) (wP)
	F	M		
4 – 9	69	90	159	100
10 – 14	61	68	129	100
15 – 19	56	38	94	100
20 – 39	223	202	425	91
40 – 59	246	219	465	71
≥ 60	109	119	228	64
Total	**764**	**736**	**1,500**	**83%**

HU/USP, Hospital Universitário/Universidade de São Paulo.

As for the vaccination status, it was not possible to verify the vaccination records of all volunteers, however parents or guardians, referring to the age groups 4-9, 10-14 and 15-19 years, when filling out the questionnaire on vaccination and health status, unanimously stated that all vaccines provided for in the Brazilian vaccination schedule were up to date, including DTwP (diphtheriae-tetanus-pertussis) vaccine. Percentage of fully immunized individuals decreased with increase in age groups reaching 91% (386/425), 71% (331/465) and 64% (146/228) in the age groups 20-39, 40-59 and ≥ 60 y, respectively (**Table 1**).

### 2.2 Ethics

This study was approved by the University of São Paulo’s Hospital Committee for Ethics in Humans (Plataforma Brasil – CAAE - 3687114.8.3001.0076; Protocol: 728400). All human blood samples were collected with their consent or the consent of the respective guardian.

The animal experiments were approved by the Committee for Ethics in Animal Use at Instituto Butantan (CEUAIB, Protocol 3469110820). Animal experiments complied with the ARRIVE guideline.

### 2.3 ELISA for human and mouse studies 

**Supplementary Protocol 1** - in summary, 96-well plates (Nunc MaxiSorp^®^) with PT (Sigma-Aldrich) (1 μg/mL) in carbonate/bicarbonate buffer (pH 9.6), incubated for 12-16 h at 4°C. After incubation, the plates were washed with wash buffer (PBS with Tween 20 0.05%) and blocked with non-fat dry skimmed milk (Molico^®^) at 10% in PBS, followed by incubation for 1 h at 37 °C. After washing the plates, serum samples were diluted in 1% PBS/bovine serum albumin (BSA) (human, dilution of 1:100 and mouse, initial dilution of 1:200) and incubated for 2 h, as described (12). After washing, the secondary antibody was incubated for 1 h: Secondary antibodies were incubated for 1 h at 37 °C: goat anti-human IgG peroxidase (Sigma-Aldrich) (1:10,000), or goat anti-mouse IgG (1:10,000), IgG1 or IgG2a (1:1,000), accordingly. The reaction was revealed by the addition of 100 µL of TMB substrate reagent (BD OptEIA™) for 15 min at room temperature, protected from light and the reaction was stopped with the addition of 50 µL of H_2_SO_4_ 4 M. The reading was performed in an ELISA reader (Gen5/Bio-Tek Instruments, Inc.), at a 450 nm wavelength. Human IgG values were converted into IU/mL using a standard curve with Pertussis Antiserum (human) 1st IS-WHO international Standard (NIBSC 06/140) with 335 IU per ampoule. Similarly, using an initial dilution of 1:20 in 1% PBS/BSA, serum samples were incubated as above, to evaluate human anti-PT IgM. No conversion was used for IgM due the absence of a standard curve and values were plotted as absorbance at 450 nm at the 1:20 dilution.

### 2.4 Cytokine measurement in whole blood human samples

Human whole blood stimulation was performed as previously described (wP, n=784; Plow, n=753) (13). Briefly, whole blood stimulation was performed by diluting 250 μL of fresh heparinized human whole blood (containing ~ 1.8 × 10^6^ nucleated cells) in 400 μL of RPMI-1640 medium (GIBCO^®^, Life Technologies, Paisley, UK) in the presence of 250 μL of the stimuli (wP vaccine or Plow, 10^8^ CFU), in 2 mL polypropylene tubes (Corning^®^ Inc., Corning, NY, USA). Negative control did not contain any stimuli. Positive controls received 10 μg/well of Concanavalin A (Sigma-Aldrich Inc., St. Louis, MO). Tubes were tightly capped, mixed by inversion, and incubated at 37°C for 24 or 48 h. After incubation, cells were lysed by the addition of 100 μL of 5% Triton-X and vortex. Samples frozen at −80°C until use for cytokine measurement.

Frozen samples were thawed, centrifuged, and the supernatants assayed for Th1/Th2/Th17-related cytokines (IL-2, IL-4, IL-6, IL-10, TNF-α, IFN-γ, IL-17A) with Cytometric Bead Array kits (CBA; BD Biosciences, San Jose, CA), as per the manufacturer’s instructions in a FACS Canto II flow cytometer (BD). The assays’ lower limits of detection were between 2.6 and 18.9 pg/mL, depending on the cytokine, and the higher limit was 5,000 pg/mL.

### 2.5 Bacterial strain and vaccines

*B. pertussis* (ATCC 18323) grown in Bordet–Gengou agar was used for mice challenge experiments. wP was produced by Instituto Butantan (strain 137). DTaP used was Adacel-Sanofi, composed by diphtheria toxoid (2 UI), tetanic toxoid (20 UI) and acellular Pertussis (aP 2.5 µg). Plow vaccine was developed at Instituto Butantan by chemical extraction of lipo-oligosaccharides from wP, as previously reported (11). All 3 vaccines, aP, wP and Plow were administered with 50 µg/mL of Aluminum hydroxide, Alhydrogel (Invivogen, California, USA).

### 2.6 Mouse model for prime/boost scheme

Five- to seven-week-old female BALB/c mice (5/group) were immunized intraperitoneally (i.p.) with wP (5.6 opacity units, OU) twice 2-weeks apart. Control group was injected with saline. At week 25, mice received a booster (i.p.) with wP (5.6 OU), aP (1.25 µg) or Plow (5.6 OU). Animals were bled at intervals ranging from 2-5 weeks (2^nd^, 4^th^, 9^th^, 13^th^, 16^th^, 19^th^ and 25^th^ week) to evaluate antibody decay. On week 31, spleens and lymph nodes were recovered, and single cell suspensions used for immunophenotyping and cytokine production upon stimulation.

### 2.7 Cytokine and immunophenotyping in immunized mouse splenocytes

Cytokines were determined by mouse Th1/Th2/Th17-related CBA Kit (BD) in the supernatants of single cell suspensions of splenocytes from immunized mice stimulated with PT (1 µg/mL) and incubated for 48-72 h at 37 °C. Single cell suspensions (unstimulated) were stained for T cells using anti-CD3-APC-Cy7 (clone 17A2), anti-CD4-PercP (clone RM4-5), anti-CD8-PE-Cy7 (clone 53-6.7), anti-CD44-APC (clone IM7) and anti-CD62L-FITC (clone MEL-14); or B cells: anti-B220-FITC (clone RA3-6B2), anti-CD19-BV421 (clone 1D3) and anti-CD27-PercP (LG.3A10). Cell suspensions were acquired (100,000 cells/sample) in the flow cytometer as above (**Supplementary Figure 2**).

### 2.8 Challenge and CFU count

Immunized and control mice were challenged intranasally (i.n.) with *B. pertussis* (10^7^ CFU in 40 µL of PBS) and euthanized one week later to determine CFU count. The lung was homogenized using a tissue grinder with PBS and serial dilutions plated in Bordet-Gengou Agar and incubated at 37°C for 5 days. Bronchoalveolar fluid (BAL) was collected as previously described (14) and used to determine CFU, cell count and cytokine production. Briefly, after euthanasia, each animal trachea was exposed, cannulated, and a syringe used to inject 0.5 mL of cold PBS into the bronchoalveolar space and collected. Then, another 1 mL of cold PBS was injected, rinsed 3 times, and collected. BAL was centrifuged at 5,000 x g, 4°C for 15 min for differential leukocyte count. Briefly, 200 μL of BAL suspension (~ 4 x 10^4^ cells) were centrifuged at 27 x g for 4 min in the cytocentrifuge (StatSpin Cytofuge, Beckman Coulter) to obtain the slides. After drying, an “INSTANT PROV” (Interlab) cell staining kit for quick differential coloring was used.

The BAL supernatants were stored at -80°C to the assessment of Th1/Th2/Th17 cytokines by Cytometric Bead Array – Th1/Th2/Th17 kit, CBA Kit (BD), following the manufacturer´s recommendations.

### 2.9 Statistical analysis

Human seroprevalence data: standard curve of NIBSC 06/140 serum combined a 4PL (4-parameter logistic regression) and linear regression to better predict lower antibody levels. One-way ANOVA followed by Tukey’s post-test (GraphPad Prism 8) were used for differences between antibody levels and cytokine secretion of human blood samples. Mouse experiments: Antibody levels, memory B cells and CFU counts were compared by Mann-Whitney U-test.

## 3 Results

### 3.1 Seroprevalence of anti-PT IgG and IgM across age groups

This study assessed anti-PT IgG in 1,500 and anti-PT IgM in 1,024 blood samples collected between 2015 and 2018, from healthy donors ranging from 4-76 years of age, which were stratified into six age groups (**Table 1**). The median age of participants was 36.9 y with 50.9% (764) female and 49.1% (736) male participants. All volunteers < 19 y of age declared strict compliances to the vaccination schedule, while for those > 19 y compliance gradually decreased with age (**Table 1**).

**Figure 1:**
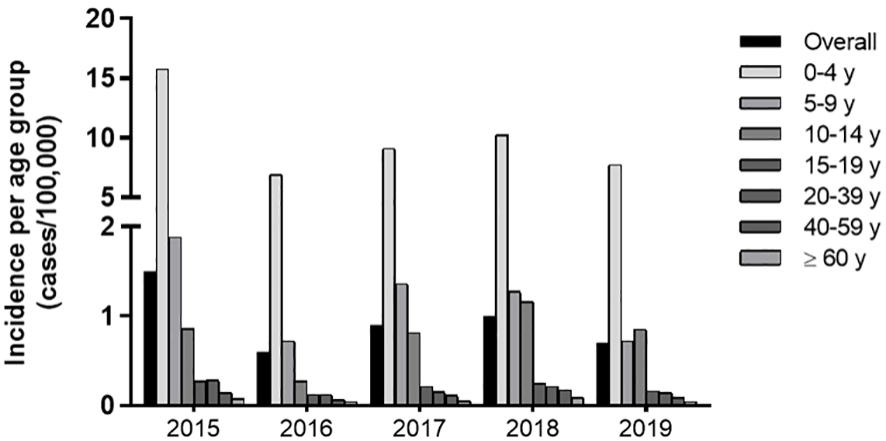
Incidence of Pertussis (2015-2018) in Brazil per age group. Incidence of Pertussis defined by official reported cases from 2015-2018 according to the SINAN/DATASUS database and IBGE data on population.

Historically, reported cases of pertussis are higher in children below 4 y, which accounted for 74% of the total cases reported. Between 2015 and 2018, incidence is this age group ranged from 15.8 to 6.9 and gradually decreased with age (**Supplementary Table 1**). The 5-9 y group exhibits a 1.9-0.7 and the 10-14 y, a 0.8-0.3 incidence. Above 15 y incidence falls to 0.3-0.1 cases per 100,000 individuals (**Figure 1**; **Supplementary Table 1**).

**Figure 2:**
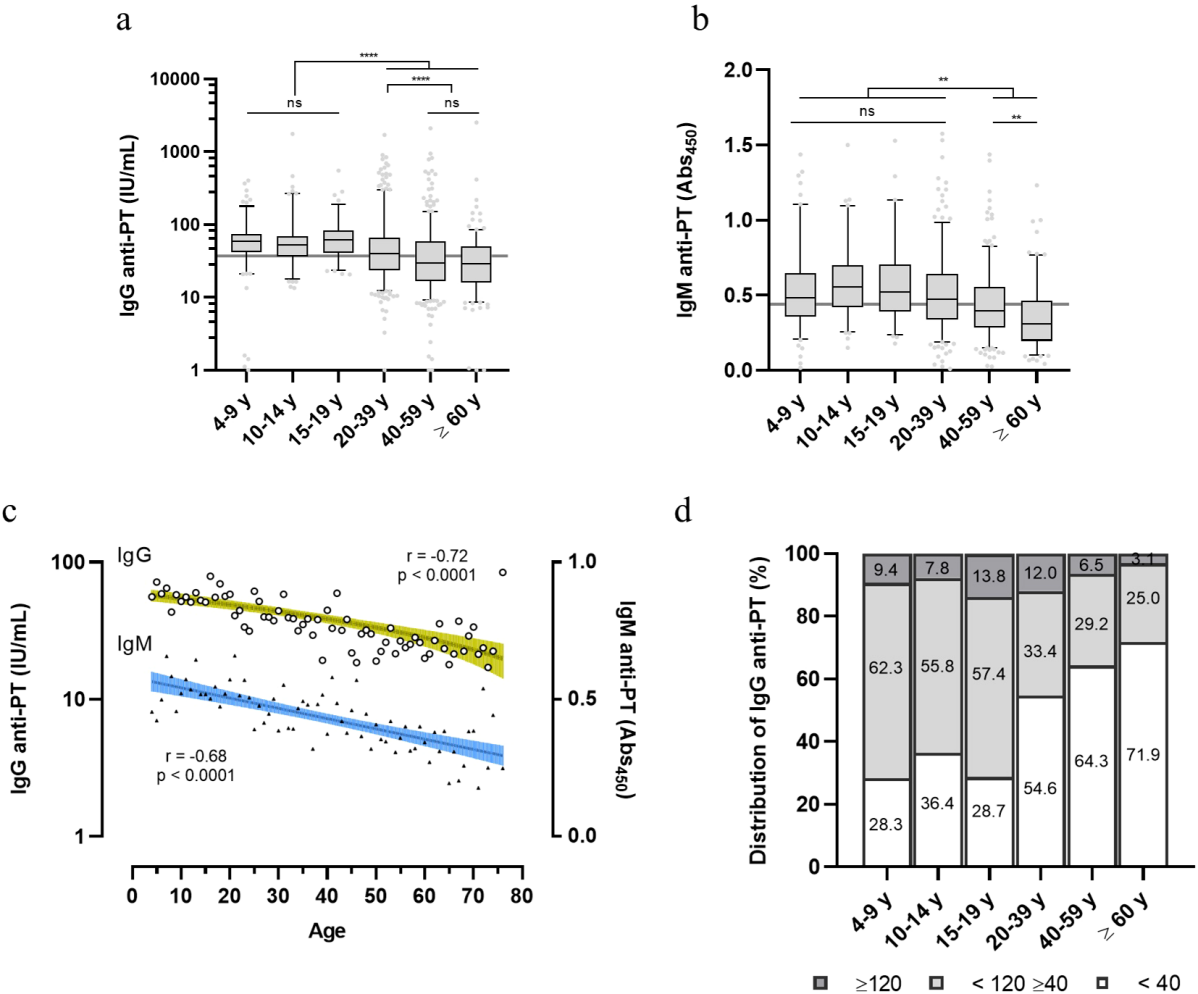
Distribution of anti-PT IgG and IgM within healthy volunteers in Brazil. Anti-PT **(A)** IgG (IU/mL) and **(B)** IgM (Abs_450_) detected in the sera of healthy Brazilian donors are represented as boxes and whiskers represent value distribution with median (horizontal line), first, and third quartile (box limits) and 5-95 percentiles (whiskers). Overall median of the data is presented as a grey line underneath the data. **(C)** Pearson’s correlation of IgG and IgM data according to age. Linear regression and 95% confidence bands (colored green and blue bars, respectively). **(D)** Distribution of the IgG levels within the age groups categorized according to seroprevalence standards for infection/recent contact; past contact and negative (≥ 120, dark grey; <120 ≥ 40, light grey; and < 40 IU/mL, white bars, respectively). Values inside bars indicate the percentage of samples in each category. One-way ANOVA with Tukey’s post-test was used to determine statistical significance (p-values ≤ 0.05) of the difference between groups. **p < 0.01, **p < 0.005, ****p < 0.0001 and ns = not significant.

Diagnostic levels of serological anti-PT antibody that have been assigned represent either: no recent exposure to vaccine or infection (< 40 IU/mL), indication of exposure or vaccination in the last year (>40 and < 120 IU/mL) or recent exposure (>120 IU/mL) (15–17). Anti-PT data on IgG grouped accordingly shows that the younger population (4-9, 10-14 and 15-19 y) exhibits higher anti-PT IgG levels as compared to the adult and elderly population (20-39, 40-59 and ≥ 60 y), (>40 and < 120 IU/mL) (**Figure 2A**). The young adult group (20-39 y) also exhibits higher anti-PT IgG levels than older adults, in the same anti-PT antibody range. Confirming these results, anti-PT IgM values showed a similar pattern, where the younger age groups (up to the 20-39 y) exhibit a higher IgM reactivity than older adults and the elderly (**Figure 2B**). Therefore, since the last booster dose is at 4-5 y and immunity is considered to last between 5-10 y, the groups of 15-19 y and 20-39 y, should have had some exposure to *B. pertussis*. Full data on IgG and IgM per age group are available in **Supplementary Table 2**. Pearson’s correlation demonstrates that both anti-PT IgG and IgM decayed with age (**Figure 2C**). Indications of a recent exposure (≥ 120 IU/mL) were detected in 8.4% of the population sample; the age groups between 15-39 y showed the highest percentage of positive samples (13.8-12.0%) (**Figure 2D**). Indications of exposure in the last year (≥ 40 and <120) were mainly present in the younger population (57.4-62.3%). Over half of the donors (54.3%) exhibit anti-PT IgG levels under 40 IU/mL, considered to be negative for exposure (**Figure 2D**).

There was a negative correlation (p < 0.001) associated with the anti-PT levels as an indicator of recent pertussis contact (≥ 40 < 120 IU/mL) and the group of adolescents and young adults (26, IQR 12-46 years); so do contact with children. Additionally, smoking and chronic pulmonary diseases were also negatively correlated (**Table 2**). Chronic diseases such as high pressure, cardiopathy, diabetes, asthma, bronchitis, etc, were identified in 644 out of 1,500 volunteers. Furthermore, higher anti-PT IgG levels are associated with increased IL-2, and lower IL-6 and IL-10 (p < 0.001).

**Table 2:** Demographic variables and cytokines associated with the indication of positive seroprevalence for anti-PT IgG.

Variable	Overall n = 1,500	Less than 40 IU/mL n = 765	Between 40 and 120 IU/mL n = 615	Greater than 120 IU/mL n = 120	p-value*^1^*
Age, Median (IQR)	37 (19 – 53)	46 (29 – 57)	26 (12 – 46)	28 (17 – 42)	<0.001
Sex, n (%)					0.082
Female	764 (51)	368 (48)	331 (54)	65 (54)	
Male	736 (49)	397 (52)	284 (46)	55 (46)	
Vaccine, n (%)					0.43
Yes	1,245 (83)	637 (83)	499 (81)	109 (91)	
Do not know	240 (16)	125 (16)	105 (17)	10 (8.3)	
No	15 (1.0)	3 (0.4)	11 (1.8)	1 (0.8)	
Has had whooping cough, n (%)	96 (6.4)	63 (8.2)	26 (4.2)	7 (5.8)	0.01
Has contact with children, n (%)	826 (55)	388 (51)	372 (60)	66 (55)	0,001
Smoker, n (%)					<0.001
Never	1,110 (74)	494 (65)	507 (82)	109 (91)	
Previously	261 (17)	181 (24)	73 (12)	7 (5.8)	
Yes	129 (8.6)	90 (12)	35 (5.7)	4 (3.3)	
Chronic disease, n (%)	644 (43)	387 (51)	219 (36)	38 (32)	<0.001
Cytokines^2^, Median (IQR)					
IL-2	1.6 (0.0 – 4.4)	1.3 (0.0 – 3.2)	2.1 (0.2 – 6.1)	1.7 (0.0 – 5.1)	<0.001
IL-4	0.07 (0.00 – 1.33)	0.06 (0.00 – 1.23)	0.13 (0.00 – 1.49)	0.00 (0.00 – 1.29)	0.37
IL-6	1,490 (540 – 4,214)	1,900 (677 – 5,672)	1,259 (414 – 2,986)	1,340 (698 – 2,862)	<0.001
IFN-γ	2 (0 – 8)	2 (0 – 7)	3 (0 – 10)	1 (0 – 8)	0.19
TNF-α	0.27 (0.00 – 2.48)	0.45 (0.00 – 2.47)	0.19 (0.00 – 2.58)	0.00 (0.00 – 2.15)	0.6
IL-17	0 (0 – 7)	0 (0 – 6)	0 (0 – 9)	0 (0 – 6)	0.63
IL-10	1.4 (0.3 – 4.2)	1.6 (0.5 – 4.6)	1.2 (0.0 – 3.6)	1.0 (0.0 – 2.8)	0.008

*^1^* Kruskal-Wallis rank sum test; Pearson’s Chi-squared test.

*^2^* ng/mL. Missing data. Overall (n = 733), Less than 40 (n=366), Between 40 and 120 (n = 314), Greater than 120 (n = 53).

### 3.2 Cellular immune responses from stimulated human blood samples

**Figure 3:**
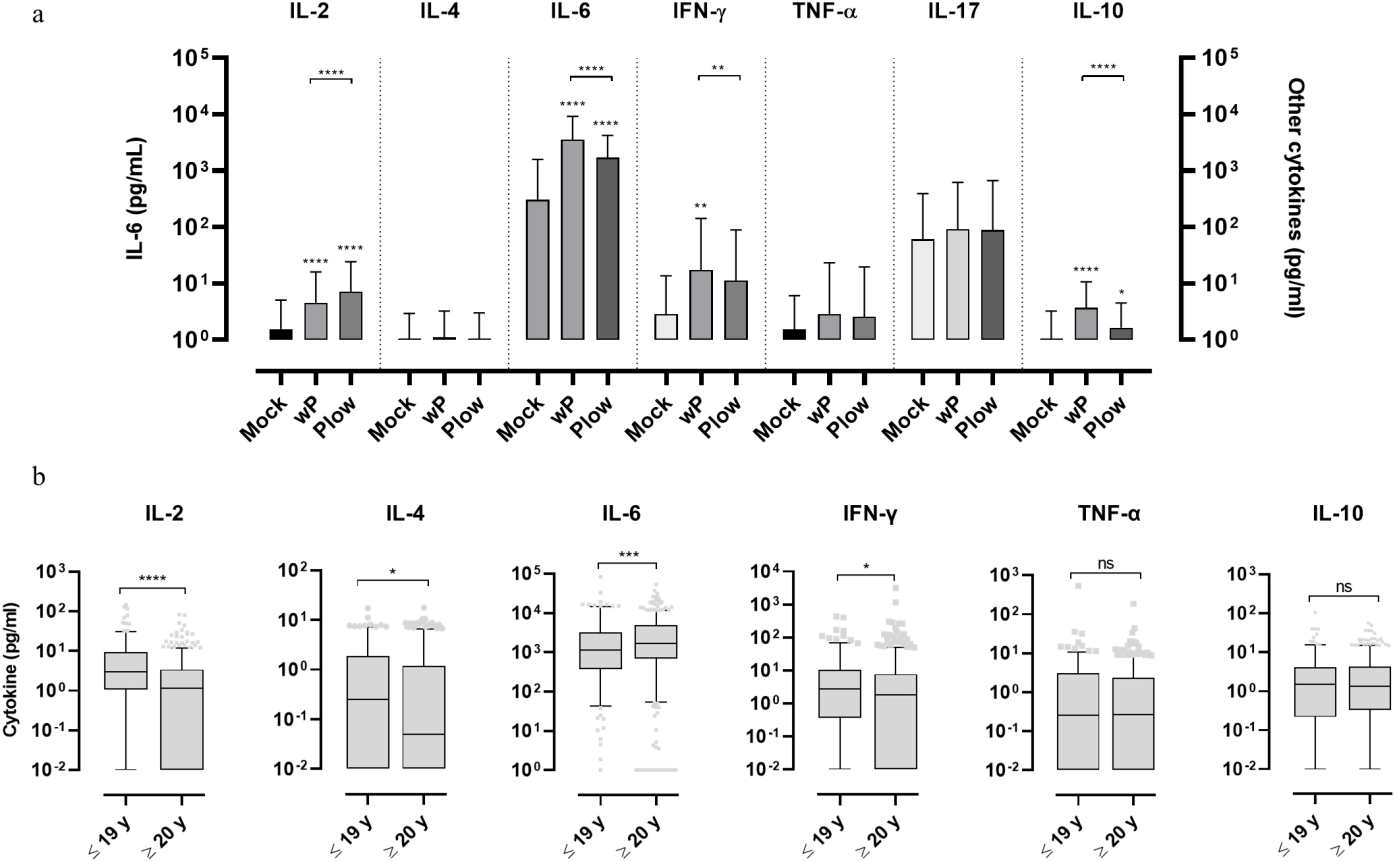
Cytokine response of whole blood cells from volunteers in response to stimulation with wP or Plow vaccines. **(A)** Blood samples from volunteers were stimulated *in vitro* with wP or Plow vaccines (10^8^ cells/mL). After 24-48 h, supernatants were collected to measure cytokine concentrations (IL-2, IL-4, IL-6, IFN-γ, TNF-α, IL-17A and IL-10) by flow cytometry (CBA kit). Values are represented as symbols at the median and confidence intervals (95% CI). One-way ANOVA with Tukey’s post-test was used to determine statistical significance (p-values ≤ 0.05) of the difference between groups *p ≤ 0.05, **p < 0.01, ****p < 0.0001. **(B)** The data was segregated into different age groups (pool of ≤ 19 y and pool of ≥ 20 y) stimulated *in vitro* with wP vaccine. Bars represent median and 95% CI. Mann-Whitney U-test was used to determine statistical significance of the difference between groups. *p ≤ 0.05, ***p < 0.001 and ****p < 0.0001, ns, not significant. Data on IL-17 resulted in very low levels and were, therefore, excluded from the analysis.

To comparatively determine the potential human leukocyte activation whole blood cells were cultured and stimulated with wP or Plow. Both vaccines showed higher responses of IL-2, IL-6, IFN-γ and IL-10 in comparison to the unstimulated control; production of IL-6 was by far the highest (**Figure 3A**). There were also differences between cytokine production following stimulation with either wP or Plow, which could be attributed to its lower LPS content. While IL-2 was greater in Plow-stimulated cells, IL-6, IFN-γ and IL-10 levels were decreased. When the samples were classified as to the age of donors, both wP and Plow, independent of age, were able to stimulate the secretion of IL-6 (**Supplementary Figure 3A**). Full data on cytokine levels per age group are available in **Supplementary Table 2**. No correlation was found between the cytokine responses and antibody levels (**Supplementary Figure 3B**).

Whole blood cells of all the 1,500 samples, cultured and stimulated with wP, showed higher Th1 (IL-2 and IFN-γ) and Th2 (IL-4) cytokines in the ≤ 19 y group than the ≥ 20 y group (**Figure 3B**). This separation (below and above 19-20 y) was defined based on the assumption that the younger age groups which exhibit higher antibody levels would also show a higher response from circulating leukocytes upon wP stimulation. IL-6 was also higher in the ≥ 20 y group. No differences were observed in TNF-α, IL-10 or IL-17 levels.

### 3.3 Waning mouse immunity and booster vaccine effects

**Figure 4:**
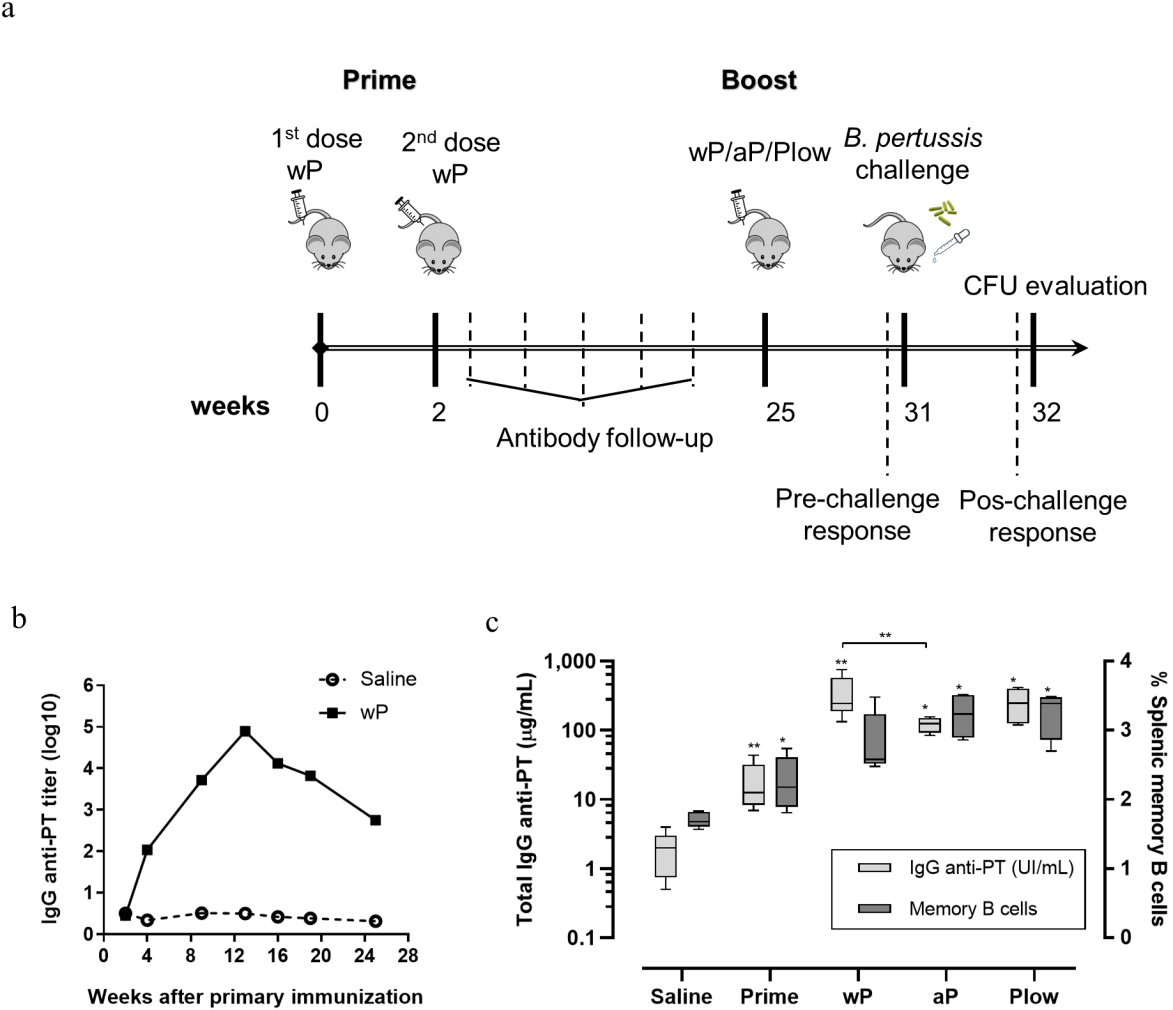
Humoral and memory B cell response elicited in prime/boost immunization in the mouse model. **(A)** Schematic representation of the immunization of mice with wP prime and boost with different vaccines. Groups of mice (10 per group) were immunized twice with wP in a two-week interval. Serum anti-PT IgG was followed until decay. At the 25th week a boost dose of wP, aP or Plow was administered, and immune response evaluated 6 weeks later (31^st^ week) (5 per group) or challenged with *B*. *pertussis* and evaluated for CFU in BAL and lungs (5 per group). **(B)** Follow-up of anti-PT IgG (log10 titers) in the sera (pool of the group) of mice primed with two doses of wP or not immunized (Saline). **(C)** Determination of seric anti-PT IgG levels (light grey boxes) and memory B cells (dark grey boxes) in the spleen before (Prime) and after boost with wP, aP or Plow. Boxes and whiskers represent value distribution with median (horizontal line), first, and third quartile (box limits) and minimum and maximum values (whiskers). Mann-Whitney U-test was used to determine statistical significance of the difference between groups. *p ≤ 0.05 and **p < 0.01. Asterisks above boxes of wP, aP and Plow groups represent the comparison with the Prime group.

If immunity to pertussis wanes with time and adolescents/young adults are the major groups from which pertussis could transmit to susceptible hosts, we resorted to the mouse model to question whether a booster could restore immunity and protection. Mice were immunized with wP and then boosted during waning immunity with wP, aP, or Plow (**Figure 4A**). The peak of anti-PT antibody occurred at 12 weeks following the first dose, then declined two log_10_ by 24 weeks (**Figure 4B**). All booster doses significantly increased antibody levels, with wP inducing higher levels of anti-PT antibodies than aP (**Figure 4C**). Splenic memory B cells were present after priming and further increased by aP/wP or Plow boosts (**Figure 4C**).

**Figure 5:**
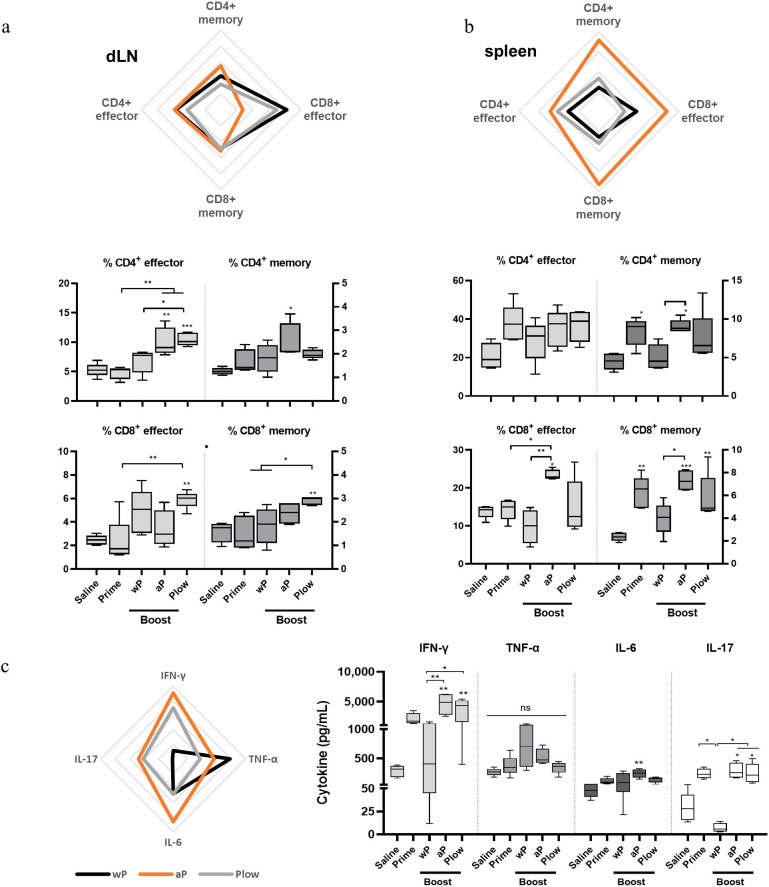
Local and systemic cellular immune response profile/Effector and central memory (TCM) CD4^+^/CD8^+^ T cells induced in prime/boost immunized mice. T cells from **(A)** the draining lymph nodes (dLN) and **(B)** spleens of immunized mice (5 per group) were characterized as to CD4^+^ or CD8^+^ effector cells (CD3^+^CD44^+^CD62L^-^) or central memory T cells (TCM) (CD3^+^CD44^+^CD62L^+^) phenotypes. Overall visualization of cellular phenotypes of the groups receiving boost in the radar charts were: wP, black line; aP, orange line; Plow, grey line). Values were converted to fold-change over the Prime group. Each line of the radar graphs represents a 0.5-fold increase. Percentages of effector and central memory T cells of all groups are shown as boxes and whiskers represent value distribution with median (horizontal line), first, and third quartile (box limits) and minimum and maximum values (whiskers). **(C)** Splenocytes from immunized mice were stimulated for 48 h (TNF-α, IL-6) and 72 h (IFN-γ, IL-17) and supernatants assessed for cytokines using the CBA kit. Data on IL-2, IL-4 and IL-10 resulted in very low levels and were, therefore, excluded from the analysis. One-way ANOVA with Tukey’s post-test was used to determine statistical significance of the difference between groups. *p ≤ 0.05, **p < 0.01 and ***p < 0.001. Asterisks above symbols represent the comparison with the control Saline.

Since the duration of immunity is dependent on the formation of a memory response, we investigated the presence of effector T cells and central memory T cells (TCM) in the different vaccination schemes. Overall, effector T cells and TCM, wP and Plow had comparable effects, while aP exhibited a more distinct profile. In dLN, a boost with cellular vaccines (wP or Plow) preferentially induced effector CD8^+^ cells, while aP induced increased CD4^+^ memory cells (**Figure 5A**). In the spleens, aP strikingly increased both memory and effector CD4^+^ and CD8^+^ cells (**Figure 5B**). Cytokine profile of splenocytes from aP boosted animals stimulated *in vitro* with PT resemble that induced by Plow (**Figure 5C**). Boost with wP decreased IFN-γ and IL-17 production. Comparison of the boosted groups shows clearly that aP and Plow displayed increased IFN-γ and IL-17 production (**Figure 5C**).

### 3.4 Protection induced against challenge

**Figure 6:**
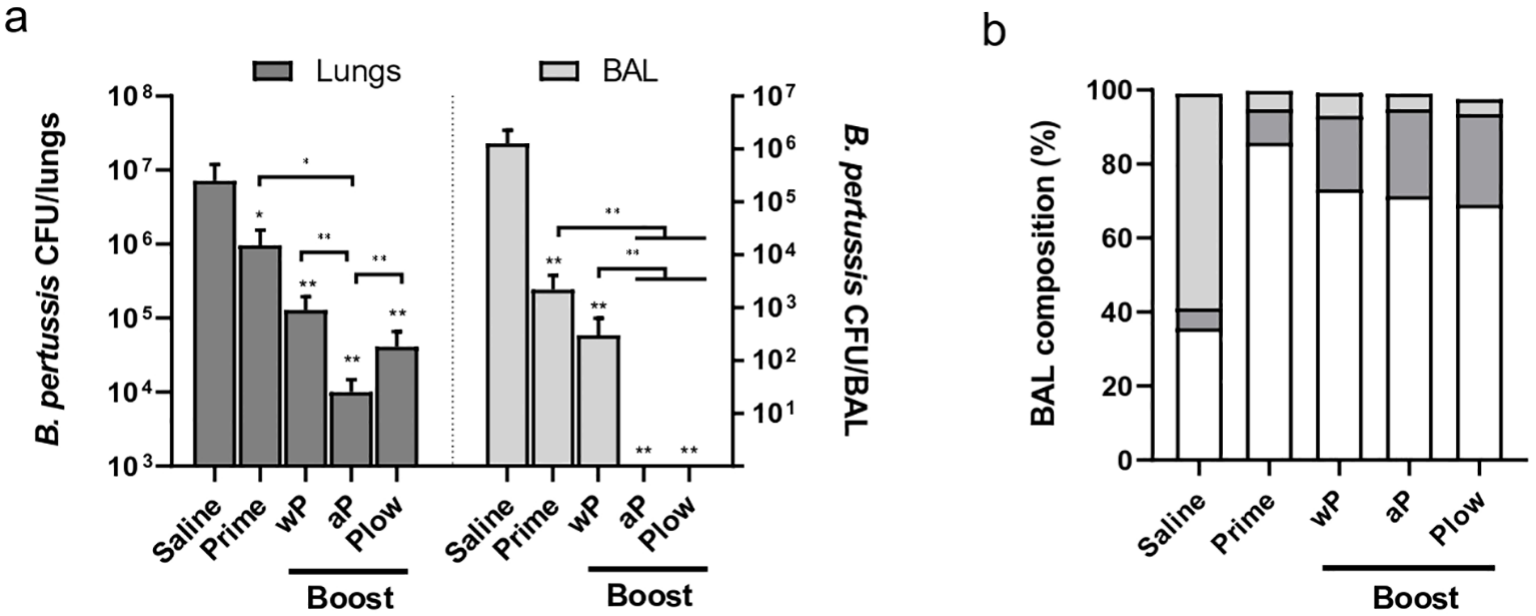
Protection induced by prime/boost immunization against *B*. *pertussis* challenge. Groups of immunized mice (5 per group) were challenged i.n. with *B*. *pertussis*. Lungs and bronchoalveolar lavage (BAL) were recovered one week later for **(A)** CFU count in the lungs and BAL (dark and light grey bars, respectively), or **(B)** determination of BAL composition as to the presence of neutrophils (light grey), lymphocytes (dark grey), and monocytes (white). Mann-Whitney U-test was used to determine statistical significance of the difference between groups. Bars represent mean ± SD of 5 mice per group. Asterisks above bars represent comparison with the control (Saline). *p < 0.05 and **p < 0.01.

All immunized groups showed reduced *B. pertussis* recovery following challenge in both the lungs and BAL as compared to the control (**Figure 6A**). Boosting with aP consistently enhanced protection compared to Prime or other boost groups. Plow also showed reduced CFU recovery, more evident in the BAL. The challenge itself (Saline) triggered mainly a neutrophilic response in the lungs/bronchoalveolar lavage (BAL). All boosted groups exhibit increased lymphocyte recruitment after challenge with *B. pertussis* as compared to Saline (**Figure 6B**, **Supplementary Figure 3A**). Pathogen clearance results in lower cytokine concentration at the target site (**Supplementary Figure 3B**).

## 4 Discussion

Pertussis remains a global public health issue. In Brazil, with historically high vaccine coverage rates, a recent peak in 2014 demonstrates pertussis’ ability to rapidly spread in the population (3). While infants are by far the most affected age group, adolescents and adults are not perceived as having an important burden.

Seroprevalence studies in the adult population commonly identify a huge distance between the officially reported cases of pertussis and anti-PT IgG levels ≥ 120 IU/mL (indicative of an acute or very recent infection) (15, 16). Our data suggests that pertussis circulates especially in schoolchildren and adolescents, but adolescents (15-19 y) along with young adults (20–39) have the highest percentage of recent exposure. Accordingly, the parents, especially mothers, are reportedly the main sources of transmission to infants, not grandparents nor the community (18). By our parameters, 120/1,500 individuals exhibit antibody levels above the threshold. Even if we disregard the possible effect of immunization and only consider age groups beyond adolescents, this greatly exceeds all-age reported rates in Brazil. Many studies in different countries also describe similar underreporting, providing strong evidence of widespread pertussis among young adults (19–23).

It has been shown that cytokines associated with a Th1 response are important for the long-term protection against *B. pertussis* after infection or immunization (24). Our results show that whole blood cells from the total cohort produce Th1 and inflammatory cytokines (IL-2, IFN-γ and IL-6) upon stimulation with either wP or Plow. Interestingly, Plow stimulated lower production of IL-6, but higher IL-2, suggesting reduction in inflammatory response, but increase in cell activation. When stratified by age, there is a clear reduction in IFN-γ and IL-2 in individuals > 20 y as compared to those < 19 y, suggesting a reduction in protective immune responses. IL-4 production is also reduced with age, suggesting an association with the lower levels of anti-PT IgG. The higher levels of IL-6 production may reflect the increase in exposure, since IL-6 is produced as part of the acute inflammatory response, recruiting leukocytes, and inducing the production of other T cell cytokines. It is interesting that previous work has shown that infants immunized with DTaP vaccine have an overall lower cellular response than adults (25). This divergence can be due to the fact that in Brazil, infants are immunized mostly with the DTwP vaccine.

If pursuing cocooning strategies to protect individuals at risk by vaccinating those in close contact, which vaccine should be used to boost young adults? To mimic waning immunity, we immunized mice with wP and waited until anti-PT IgG antibody titers declined approximately 2-fold. We then evaluated the ability of three different vaccines (wP, aP, and Plow) as boosters, to restore anti-pertussis immunity and protection. Although aP is known to elicit higher antibody responses than wP (27), in our mouse model wP, aP and Plow as boosters increased antibody levels 20, 8 and 15-fold, respectively. This divergence may reflect vaccine composition; here, the dose for aP was 1.25 µg of PT antigen. Other studies comparing immune responses in children (aP vs wP), used an aP vaccine with a composition containing 4 times more PT antigen (28). Accordingly, while the primed mice maintained memory B cells, boosting with aP or Plow further increased them, unlike wP booster. This indicates the potential benefit of boosting with a different vaccine. While previous research associated splenic memory B cells with wP vaccination (29), our study observed better responses with aP and Plow boosters. This difference may be attributed to the prime/boost regimen. Weaver and cols (29) used a homologous prime/boost strategy (using the same vaccine for both prime and boost), whereas our study combined wP with homologous or heterologous boost doses (using the same or different vaccines, respectively). This approach better reflects the situation in Brazil, as wP is used in primary immunization. aP is only given to special groups such as pregnant women and other risk groups. In fact, it is reported that the combination of vaccines that include at least one dose of wP is sufficient to induce a broad B cell response (30).

Only a boost with aP or Plow increased CD4^+^ and/or CD8^+^ in dLN and/or spleen, confirming the beneficial effect of combining different types of vaccines. While effector T cells are important at the acute phase of the disease, the induction of long-lived memory cells are ideal for sustained immunity. Accordingly, in stimulated splenocytes, aP and Plow induced an increase in IFN-γ and IL-17, associated with Th1/Th17 responses. The Th1/Th17 response is usually associated with wP immunization (or infection), while aP is associated with a Th2 profile. Our results reinforce the idea of a Th1/Th17 imprinting when wP is used as priming even with repeated aP boosters (26). Recent work demonstrated the critical role of IL-17 in both natural and acquired immunity against pertussis (31). The prime-boost regimen using aP vaccines successfully induces protection to the lungs but fails to inhibit nasal colonization of *B. pertussis*, due to the lack of Th17 CD4^+^ T cells, resident T cells in the nasal mucosa (32). The combination of wP with aP could remedy this by eliciting critical Th17 immunity thus preventing nasal colonization and, therefore, evolution of the infection to the lower respiratory tract. A further benefit would be the production of antibody-mediated immunity through opsonization, phagocytosis and complement-mediated killing, as demonstrated by previous studies (27).

All boosted groups displayed improved control of pathogen dissemination upon B. pertussis challenge, with aP or Plow boosters showing particularly strong results. These findings suggest that while homologous wP boost may primarily act through antibody-mediated responses (given the minimal induction of memory B cells, effector/memory T cells or cytokines) heterologous boosting with aP or Plow enhances immunogenicity and protection. This improvement could be due to expanding memory B cells, effector and/or memory T cells in the lymphoid organs, or Th1/Th17 responses. Interestingly, the boosted groups exhibited a more pronounced monocytic and lymphocytic influx upon challenge, indicating recognition of the recent antigenic stimulation. In contrast, the predominant neutrophil influx observed in unimmunized mice reflects a less specific immune response.

We acknowledge limitations in our study and caution should be exercised in the interpretation of the results. As a limited amount of information was collected from the patients and only through an interview, information regarding health status should be interpreted cautiously. As a consequence, possible confounding factors on the IgG antibody levels may mask/skew the values as a result of e.g. drug intake or unknown infection. Additionally, the collection of blood samples was restricted to a single hospital. Even with a general attendance, this population could be of a particular community and extrapolation of underreporting may be different from other regions/hospitals. Additionally, as the donors were approached at the hospital’s outpatient care section some health concerns may be present. Another caveat is the use of BALB/c mice in the experiments. It is known that BALB/c mice skew the immune response towards Th2 responses. Other strains such as C57Bl/6 mice could exhibit airway responses to injury more similar to humans (33). On another aspect, waning immunity in the mouse experiments was based solely on the decrease of anti-PT IgG levels in the serum. Furthermore, extrapolation of mouse data to humans is uncertain.

Our study provides pertussis seroprevalence information in the Brazilian population, supporting adolescents and young adults as sources of infection to newborns. Administration of wP or aP boosts, readily available through the health care system, could be used. Despite their great effectiveness in preventing severe disease, both vaccines have limitations, potentially addressed by Plow, an alternative to the reactogenicity of wP and the higher cost of aP. In any case, given the potential role of Plow as a lower-cost alternative, future work should address immunogenicity and safety profile in larger clinical trials enrolling different age groups. Finally, our findings support the use of a booster dose (probably aP) in adolescents and young adults to reinforce immunity and prevent pertussis dissemination to infants at risk.

## Data Availability

The original contributions presented in the study are included in the article/**Supplementary Material**. Further inquiries can be directed to the corresponding author.
