# Supplementary material for: Seroprevalence study reveals pertussis underreporting in Brazil and calls for adolescent/young adult boosting: mouse model demonstrates immunity restoration
Source: Front Immunol. 2024 Dec 4;15:1472157. doi: 10.3389/fimmu.2024.1472157 (PMC11652360; doi:10.3389/fimmu.2024.1472157)
Supplement: Supplementary file 1 [file DataSheet1.pdf]

Supplementary Material

Seroprevalence assessment of pertussis in a Brazilian sample population suggests a boost in adolescents and young adults: demonstration that revaccination can restore waning immunity in mice

Eliane P. Silva[1], Monalisa Trentini[1], Dunia Rodriguez[1], Alex I. Kanno[1], Filumena M.S. Gomes[2], Maria H. Valente[2], Carlos E.M. Trufen[1&], Lais S. Yamamoto[1], Arthur D. Januzzi[1], Priscila S. Cunegundes[1], Ricardo Palacios[3#], Renan P. Souza[4], Isaías Raw[1†], Luciana C.C. Leite[1]*, Waldely O. Dias[1].

[1] Laboratório de Desenvolvimento de Vacinas, Instituto Butantan, São Paulo, SP, Brazil.

[2] Department of Pediatrics, Faculdade de Medicina da Universidade de São Paulo, São Paulo, SP, Brazil.

[&] Current affiliation, Laboratory of Immunochemistry, Prevor, Liège, Belgium.

[3] Clinical Trials Division, Instituto Butantan, São Paulo, SP, Brazil.

[#] Current affiliation, GSK Vaccines srl, Siena, Italy.

[4] Instituto de Ciências Biológicas, Universidade Federal de Minas Gerais, Belo Horizonte, MG, Brazil.

[†] In memoriam

* **Corresponding author**:

Luciana C.C. Leite, Laboratório de Desenvolvimento de Vacinas, Instituto Butantan,

Av. Vital Brasil 1500, 05503-900 São Paulo, SP, Brazil.

e-mail: luciana.leite@butantan.gov.br

telephone: +55 (11) 2627 9816

Table of contents

Supplementary Protocol 1: Detailed ELISA protocol to assess IgG anti-PT in human samples

Supplementary Table 1: Estimation of Pertussis incidence in Brazil per age group

Supplementary Table 2: IgG, IgM, and cytokine levels in the blood of volunteers according to age group

Supplementary Figure 1: Flow cytometry gating strategies to characterize the immune response in mice

Supplementary Figure 2: Correlation of age, antibodies, and cytokines within individual samples

Supplementary Figure 3: Local cellular influx and cytokine response of immunized and challenged mice

Supplementary Protocol 1: Detailed ELISA protocol to assess IgG anti-PT in human samples

Assay parameter or reagent	
Microtiter plate	Nunc MaxiSorp®
Coating buffer	0.05 M carbonate-bicarbonate (pH 9.6)
Coating	PT (Sigma-Aldrich) 1 μg/mL (100 μl/well)
Coating incubation time	Overnight (16-18 h)
Coating incubation temp (°C)	4° C
Washing solution	PBS–0.05% Tween 20 (Sigma-Aldrich)
Washing cycle	200 μl three times
Diluent	PBS-1% BSA (Sigma-Aldrich)
Blocking solution	PBS-10% skimmed milk (Molico)
Blocking incubation time	60 min
Blocking incubation temp (°C)	37° C
Serum dilution	Eight 2-fold serial dilutions
Serum incubation time	2 h
Serum incubation temp (°C)	37° C
Antibody	Goat IgG anti-human (1:10,000) (Sigma-Aldrich)
Conjugate	Peroxidase
Conjugate incubation time	1 h
Conjugate incubation temp (°C)	37° C
Substrate	TMB (100 μl/well) (Sigma-Aldrich)
Substrate incubation time	15 min
Substrate incubation temp (°C)	RT
Wavelength read (nm)	450
EQUIPMENT	EPOCH (BioTek) Microplate Spectrophotometer
Reference standard calibration	WHO 06/140 (335 IU/ml)
Controls	Blank control
Lower limit of quantitation (LOQ)	-
Software	Gen5/Bio-Tek Instruments, Inc.
Analysis (curve fit)	4PL, Linear regression

[a]PBS, phosphate-buffered saline (NaCl 137 mM, KCl 2.7 mM, Na_2HPO_4 8 mM, KH_2PO_4 1.5 mM); BSA, bovine serum albumin; PT, Pertussis Toxin; TMB, 3,3′,5,5′-tetramethylbenzidine; RT, room temperature; WHO, World Health Organization; IU, international units; 4PL, four-parameter logistic function.

Supplementary Table 1: Estimation of Pertussis incidence in Brazil per age group

Estimated population per age group/years											
**Year**	**0-4 y**	**5-9 y**	**10-14 y**	**15-19 y**	**20-29 y**	**30-39 y**	**40-49 y**	**50-59 y**	**60-69 y**	**7079 y**	**80 y +**	**Total**
2015	14,677,150	14,824,270	15,840,878	17,334,098	34,432,618	32,966,515	26,817,883	21,543,957	13,997,456	7,445,269	3,595,589	203,475,683
2016	14.715.102	14.664.912	15.577.231	17,155,625	34,379,199	33,379,628	27,225,242	22,065,236	14,534,635	7,713,027	3,746,750	205,156,587
2017	14.720.765	14.593.285	15.365.250	16,821,652	34,365,192	33,709,793	27,671,889	22,566,077	15,075,620	8,001,846	3,913,372	206,804,741
2018	14.787.544	14.537.808	15.182.038	16,439,863	34,363,369	33,958,656	28,160,337	23.040,015	15,621,191	8,314,766	4,089,313	208,494,900

Confirmed pertussis cases per age group/years											
**Year**	**<1 y**	**1-4 y**	**5-9 y**	**10-14 y**	**15-19 y**	**20-39 y**	**40-59 y**	**60-64 y**	**65-69 y**	**70-79 y**	**80 y +**	**Total**
2015	1,885	430	279	137	47	186	70	6	4	7	-	3,051
2016	828	192	105	42	20	84	28	2	5	1	2	1,309
2017	1,042	299	198	125	35	101	54	5	3	6	-	1,868
2018	1,135	380	185	176	40	142	88	8	8	5	3	2,170

Matching age groups

	**Year**	**0-4 y**	**5-9 y**	**10-15 y**	**15-19 y**	**20-39 y**	**40-59 y**	**60 y +**	**Total**
Population	2015	14,677,150	14,824,270	15,840,878	17,334,098	67,399,133	48,361,840	25,038,314	203,475,683
	2016	14,715,102	14,664,912	15,577,231	17,155,625	67,758,827	49,290,478	25,994,412	205,156,587
	2017	14,720,765	14,593,285	15,365,250	16,821,652	68,074,985	50,237,966	26,990,838	206,804,741
	2018	14,787,544	14,537,808	15,182,038	16,439,863	68,322,025	51,200,352	28,025,270	208,494,900
Pertussis cases	2015	2,315	279	137	47	186	70	17	3,051
	2016	1,020	105	42	20	84	28	10	1,309
	2017	1,341	198	125	35	101	54	14	1,868
	2018	1,515	185	176	40	142	88	24	2,170
**Incidence (cases/100,000)**	2015	15.8	1.9	0.9	0.3	0.3	0.1	0.1	1.5
	2016	6.9	0.7	0.3	0.1	0.1	0.1	0.0	0.64
	2017	9.1	1.4	0.8	0.2	0.1	0.1	0.1	0.90
	2018	10.2	1.3	1.2	0.2	0.2	0.2	0.1	1.04

Estimated population per age group/years in Brazil. Source: 2000 a 2021 – Preliminary estimations by Ministério da Saúde/SVSA/DAENT/CGIAE.
Confirmed cases of pertussis. Source: Ministério da Saúde/SINAN. Data acquired through TABNET in February 2023.

Supplementary Table 2: IgG, IgM, and cytokine levels in the blood of volunteers according to age group

	Overall	4-9 y	10-14 y	15-19 y	20-39 y	40-59 y	≥ 60 y
IgG (UI/mL)							
N samples	1,500	159	129	94	425	465	228
Median (IQR)	36·9<br>(20·3 – 72·5)	58·8<br>(41·3-73·8)	52·6<br>(36·2 - 68·6)	61·1<br>(40·4 - 82·5)	39·7<br>(23·3 -66·5)	29·8<br>(16·7 - 58·6)	28·9<br>(16 - 49·7)
IgM (Abs$_{450}$)							
N samples	1,024	125	90	65	295	298	151
Median (IQR)	0·44<br>(0·32 – 0·67)	0·48<br>(0·36 - 0·65)	0·55<br>(0·42 - 0·70)	0·52<br>(0·39 - 0·70)	0·47<br>(0·34 - 0·64)	0·39<br>(0·29 - 0·56)	0·31<br>(0·20 - 0·46)
Cytokines (ng/ml)							
N samples	767	98	71	47	219	222	110
IL-2	1·6<br>(0 – 4·4)	3·3<br>(0·9 - 7·5)	3·4<br>(1·8 - 13·2)	2·8<br>(0·8 - 8·4)	1·4<br>(0 - 4·1)	1·1<br>(0 - 3·1)	0·4<br>(0 - 2·3)
IL-4	0·1<br>(0 – 1·3)	0·5<br>(0 - 2·2)	0·1<br>(0 - 1·9)	0<br>(0 - 1·5)	0·1<br>(0 - 1·1)	0<br>(0 - 1·1)	0<br>(0 - 1·4)
IL-6	1,537<br>(574 – 4,335)	856<br>(334 – 2,276)	867<br>(291 – 4,387)	1,412<br>(563 – 3,153)	1,506<br>(528 – 3,517)	2,156<br>(883 -5,956)	1,847<br>(932 – 4,866)
IFN-	2·2<br>(0 – 8·2)	2·4<br>(0·3 - 9·1)	3·7<br>(0·6 - 13·7)	3·6<br>(0·3 - 11·1)	1·9<br>(0 - 8·6)	2·2<br>(0 - 7·7)	1·1<br>(0 - 4·6)
TNF-	0·3<br>(0 – 0·5)	0·2<br>(0 - 3·2)	0·8<br>(0 - 3·3)	0·6<br>(0 - 2·3)	0·2<br>(0 - 2·4)	0·3<br>(0 - 2·3)	0·3<br>(0 - 2·3)
IL-17	0<br>(0 – 7·3)	0<br>(0 - 5·9)	0<br>(0 - 20·7)	0<br>(0 - 7·7)	0<br>(0 - 4·6)	0<br>(0 - 12·6)	0<br>(0 - 1·2)
IL-10	1·4<br>(0·3 – 4·2)	1·5<br>(0·2 - 4·6)	1·6<br>(0·2 - 4·8)	1·2<br>(0·2 - 2·8)	1·2<br>(0·3 - 3·5)	1·4<br>(0·4 - 4·5)	1·5<br>(0·5 - 5·7)

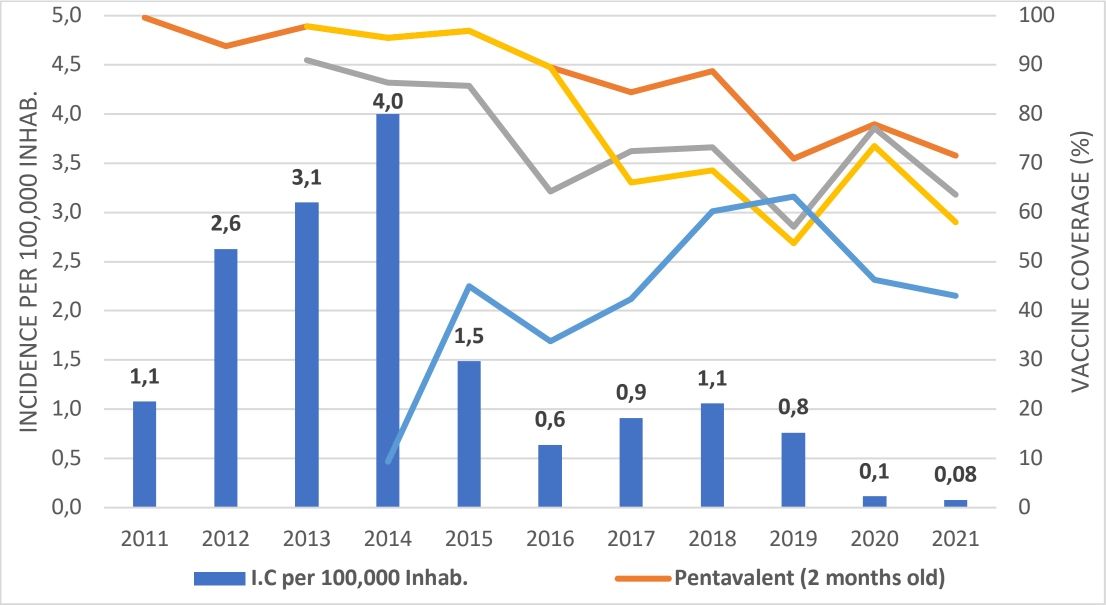

Supplementary Figure 1: Incidence Rate of the Population for Whooping Cough and Vaccination Coverage. Brazil, 2011-2021. SOURCE: TABNET/DATASUS (MINISTRY OF HEALTH, 2021); Population: IBGE/DATASUS.

**Vaccine introduced in the National Immunization Program from 2013, replacing the tetravalent vaccine.

***Vaccine introduced in the NIP

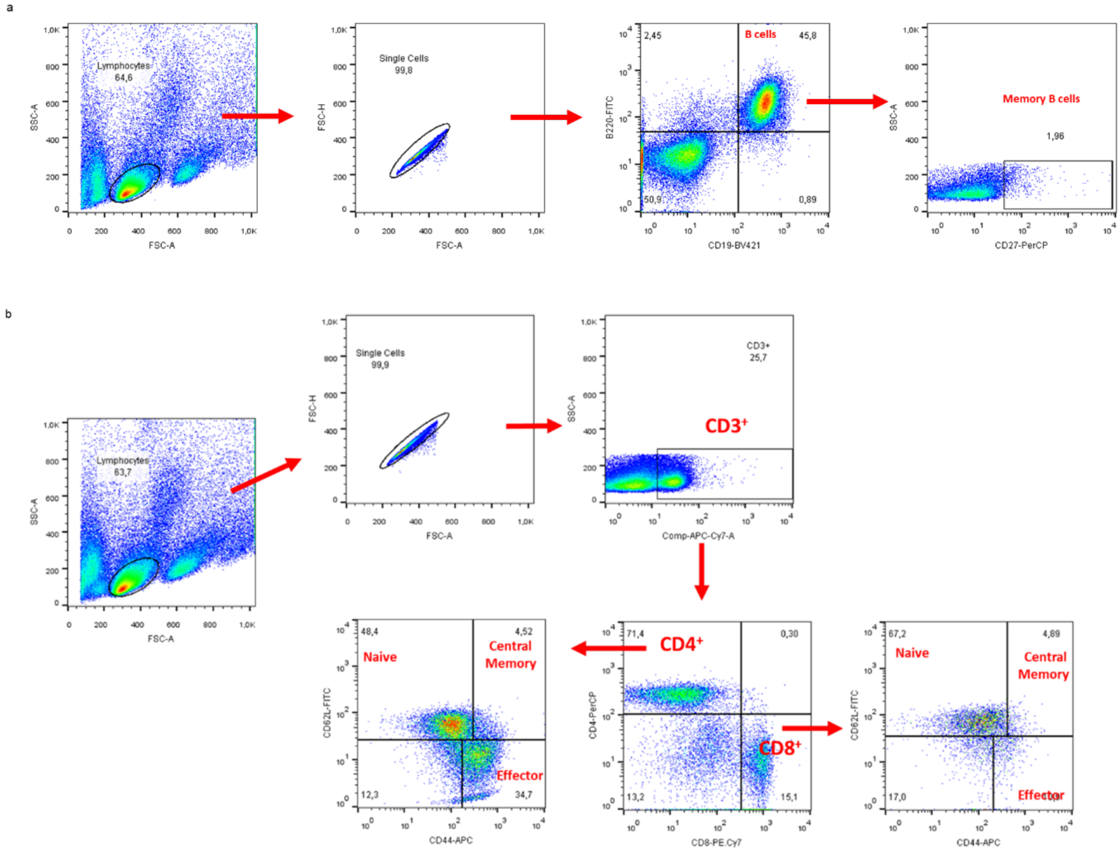

Supplementary Figure 2: Flow cytometry gating strategies to characterize the immune response in mice. Single cell suspensions of splenocytes were recovered and stained with the described markers. a) Initial gates chose lymphocytes based on side and forward scattering (SSC and FSC, respectively), single cells and then B cells ($B220^{+}CD19^{+}$). Memory B cells were gated on $CD27^{+}$ events. b) After gating of lymphocytes and single cells, $CD3^{+}$ T cells were gated on $CD4^{+}$ or $CD8^{+}$ and subsequently to $CD62L^{+}CD44^{+}$. Unstained cells were used to determine the appropriate gate of positive events. Red arrows represent the order of gating.

a

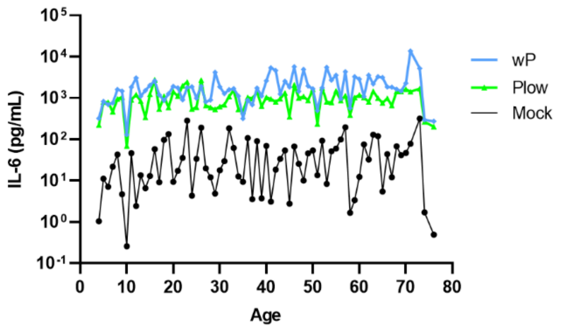

b

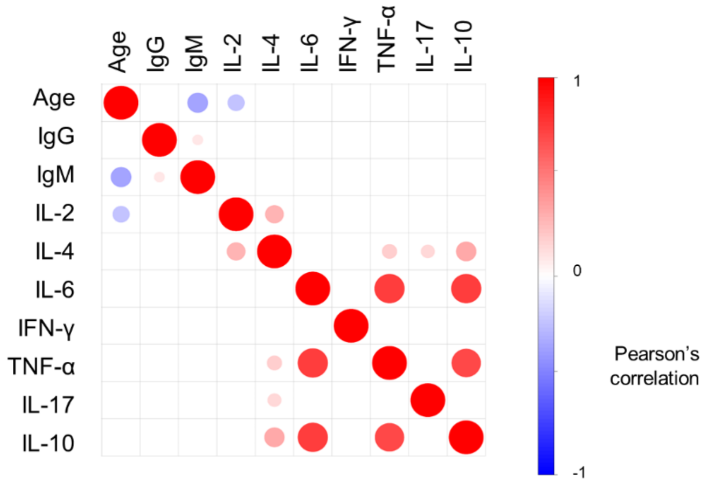

Supplementary Figure 3: Correlation of age, antibodies, and cytokines within individual samples. a) b) IL-6 levels compared according to the age of the volunteer when stimulated with wP (blue line), Plow (green line) or left unstimulated (Mock, black line). b) Paired samples that had data on IgG, IgM and cytokine secretion from whole blood cells were analysed tested using Pearson's correlation. Only statistically significant p-values are shown (circles) according to the scale (from the smallest to biggest $p \leq 0.05$, $p \leq 0.01$, $p \leq 0.001$ and $p \leq 0.0001$). Data is shown as positive (red circles, $r = 1$) and negative correlations (blue circles, $r= -1$).

a

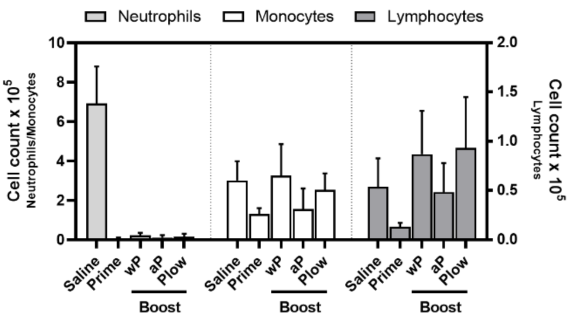

b

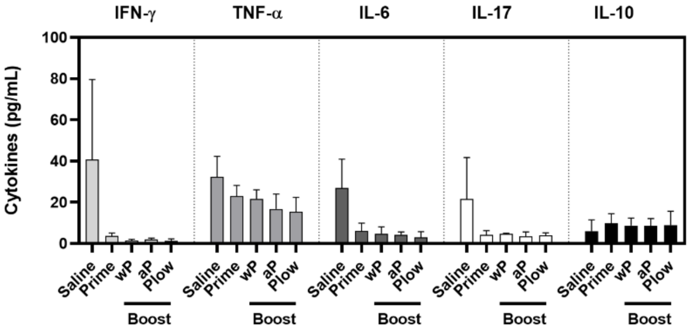

Supplementary Figure 4: Local cellular influx and cytokine response of immunized and challenged mice. After challenge, the BAL was also assessed to characterize the cellular infiltrate and cytokines present. a) Cellular influx count of neutrophils (light grey bars), monocytes (white bars) and lymphocytes (dark grey bars) in the BAL of immunized and challenged mice. b) The BAL recovered was tested for the presence of the cytokines IFN-γ, TNF-α, IL-6, IL-17, IL-10, IL-2 and IL-4. IL-2 and IL-4 resulted in levels below the detection limit and were, therefore, excluded from the analysis. Bars represent mean ± SD of 5 mice per group. One-way ANOVA with Tukey's post-test was used to determine statistical significance of the difference between groups. Asterisks above bars represent comparison with the control (Saline). * $p < 0{\cdot}05$, *** $p < 0{\cdot}001$ and, **** $p < 0{\cdot}0001$.
